# Does the Microbiota Play a Pivotal Role in the Pathogenesis of Irritable Bowel Syndrome?

**DOI:** 10.3390/jcm8111808

**Published:** 2019-10-30

**Authors:** Sharmila Fagoonee, Rinaldo Pellicano

**Affiliations:** 1 Institute of Biostructures and Bioimaging (CNR) c/o Molecular Biotechnology Center, 10126 Turin, Italy; 2 Unit of Gastroenterology, Molinette Hospital, 10123 Turin, Italy; rinaldo_pellican@hotmail.com

The microbial community that lives in the human body, called the microbiota, consists of a large variety of microorganisms including bacteria, viruses, fungi, eukaryotes and archae. Every human being harbors between 10 trillion and 100 trillion microbial cells, which is approximately equal to 10 times the total number of body cells. The term microbiome refers to the gene set of these microbial cells. The gut microbiome is estimated to contain over 150 times more genes than the human genome [1]. During long-standing interactions, a mutual co-evolution between gut microbiota and the host occurs, with the former making an important contribution to human metabolism, with significant effects on the anatomical, physiological, and immunological development of the host [2,3]. Considering that many bacterial species still cannot be cultured, our understanding of gut microbiota has evolved over the past few years thanks to the availability of advanced molecular methods that permit us to identify a large quantity of microorganisms [4]. Today, it is known that although 98% of the gut microbiota is composed of four phyla of bacteria (Firmicutes, Bacteroidetes, Proteobacteria and Actinobacteria), the majority are either Firmicutes or Bacteroidetes [4,5]. With this premise, in the last decade, the gut microbiota has become a key topic in the investigation of several gastrointestinal (GI) as well as extra-GI diseases [6,7,8,9].

Irritable bowel syndrome (IBS) is a common functional clinical condition characterized by abdominal discomfort or pain and alteration of gut habits without an underlying structural pathology [10]. As IBS affects 9–23% of the general population [11], with considerable impact on quality of life and health care resource utilization, it represents an important medical challenge in term of diagnosis and treatment [12]. The pathogenesis of IBS remains unclear, with several factors supposed to be involved, including environmental and host factors such as psychosocial stressors, food intolerance, antibiotics, enteric infections, altered pain perception, altered brain–gut interactions, dysbiosis (imbalance within the bacterial community), increased intestinal permeability, increased gut mucosal immune activation and visceral hypersensitivity [13].

In recent years, the relationship between gut microbiota and brain–gut interactions in the pathogenesis of IBS has become a key topic in clinical and biomedical research. This issue has been accurately discussed in a recent review by Quigley [14]. Beginning with an excursus on the historical steps that led to the definition of the gut–brain axis, the author discussed the modern theory that postulates the involvement of gut microbiota in both the gut–brain axis and IBS. On the basis of this theory, the pathogenesis of IBS would be related to the action of central stimuli, such as stress, that could disrupt mucosal immunity, reduce microbial diversity and alter gut barrier function, leading to gut dysfunction. Although much work still needs to be done, there is evidence to support this pathogenetic model.

First, it has been shown that fecal and colonic microbiota compositions in patients with IBS are different compared to healthy subjects. For example, the count of gene copies or the number of colony forming units of *Lactobacilli* and *Bifidobacteria* are compromised while those of *Escherichia coli* and *Enterobacter* are increased in IBS patients [15]. Furthermore, a specific gut microbiota signature (low microbial diversity, absence of *Methanobacteriales* and enrichment with *Bacteroides*) could be linked to the severity of IBS [16].

Second, the benefits reported in some studies after antibiotic or probiotic administration on IBS symptoms are proof in favor of a role of microbiota in the pathogenesis of this disease. Data from animal models provides a plausible explanation for this benefit. It has been shown that probiotics can enhance the intestinal mucosal barrier and increase the number of butyrate-producing bacteria [15,17]. The augmented production of short-chain fatty acids (SCFAs), such as butyric and acetic acid, acidizes the gut and facilitates the beneficial colonization of *Lactobacillus* and *Bifidobacterium* species [15]. Whatever the precise mechanism, the poorly absorbed antibiotic rifaximin [18] and probiotics or prebiotics [19] provided relief against the cardinal IBS symptoms. For these reasons, recent international guidelines suggest to prescribe probiotics in this context [20].

Third, the gut microbiota plays a key role in digestion, absorption and synthesis of metabolites such as SCFAs. These metabolites are directly associated with environmental factors such as diet and lifestyle [21]. Growing evidence supports the concept that, in patients with IBS, specific diets could be associated to microbiota modulation [22], thus adding another piece of the puzzle to the gut microbiota-IBS link. Of increasing interest is the low fermentable oligosaccharides, disaccharides, monosaccharides and polyols (FODMAPs) diet that leads to reduced flatulence and symptom relief in some IBS patients [23]. Nevertheless, while the beneficial role of this diet on microbiota remains unclear, a reduction of commensal bacteria has been reported. Hustoft et al. have shown a reduction in fecal *Actinobacteria*, *Bifidobacterium* and *Fecalibacterium prausnitzii* in patients on a low FODMAPs diet [24]. This warrants further investigation on the potential negative effects of “therapeutic“ diets on gut microbiota.

Fourth, the relationship between infections and IBS is well-known. It has been reported that around 10% of patients develop IBS after an infectious illness, and 3–36% of enteric infections are followed by the onset of new persistent IBS symptoms [12]. Different microbes influence the time trend features of IBS, with viral infections leading to temporary symptoms, while bacterial enteritis and protozoan and helminth infections often resulting in prolonged IBS [12]. Hypothetical pathogenetic explanations have focused on a possible residual mucosal inflammation or persistent alterations of mucosal immunocytes, enterochromaffin and mast cells, enteric nerves and the gut microbiota on the crucial role played by the immune hyperactivation [12], although further studies are needed to confirm these aspects.

Important limitations prevail in our knowledge of the relationship between gut microbiota and brain–gut interactions in patients with IBS. Among these, the role of the non-bacterial component of the gut microbiota remains unclear. For instance, whether the mycobiome (term that includes fungi and their genome) and virome have a role in the pathogenesis of stool habit alterations and in pain modulation remains to be determined [25]. The potential relationship between the type of IBS and gut microbiota also needs further investigation. Depending on predominant symptomatology, IBS is divided into four subgroups: IBS with constipation (IBS-C), IBS with diarrhea (IBS-D), IBS with a mixed pattern (IBS-M) of constipation and diarrhea, and unclassified IBS (IBS-U), without any of the previous symptoms [12]. It is not clear if a specific gut microbiota signature for each subtype exists. Considering the data available on patients with a single symptom [26], it is not astonishing to hypothesize that each gut habit could have a specific dysbiosis. Regarding IBS patients, in the meta-analysis conducted by Wang et al., among the 23 selected studies, the subtype was reported only in 15. Furthermore, small sample sizes and the heterogeneity of these studies did not allow them to draw a significant conclusion [15].

In conclusion, the review published by Quigley explores the essence of the relationship between the gut–brain axis and the microbiome and provides indications for future studies aiming at searching for novel therapeutic strategies in patients with IBS. Thus, understanding whether the gut microbiota has a role in the pathogenesis of IBS, and if the management of dysbiosis could be beneficial for these patients, remains an important issue and may provide the key to (at least partially) resolve the widespread epidemic of IBS.
